# New global climate actions: insight from COP29

**DOI:** 10.1093/nsr/nwae475

**Published:** 2025-01-08

**Authors:** Tong Jiang, Buda Su, Zbigniew W Kundzewicz, Weijie Zhao

**Affiliations:** Research Institute of Climatic and Environmental Governance/Institute for Disaster Risk Management, School of Geographical Science, Nanjing University of Information Science & Technology, China; Laboratory for Climate Risk and Urban-Rural Smart Governance, School of Geography, Jiangsu Second Normal University, China; Research Institute of Climatic and Environmental Governance/Institute for Disaster Risk Management, School of Geographical Science, Nanjing University of Information Science & Technology, China; Laboratory for Climate Risk and Urban-Rural Smart Governance, School of Geography, Jiangsu Second Normal University, China; Department of Environmental Engineering and Mechanical Engineering, Poznan University of Life Sciences, Poland; National Science Review editorial office, China

## Abstract

The COP29 held in Baku (Azerbaijan) from 11 to 24 November 2024 passed a new climate finance goal, aiming to support the developing countries to address the climate challenges.

The 29th Conference of Parties (COP29) of the United Nations Framework Convention on Climate Change (UNFCCC) was held in Baku (Azerbaijan) from 11 to 24 November 2024. This conference, gathering more than 66 700 participants from around 200 countries, came in a moment when climate change and its impacts attracted considerable attention, worldwide.

**Figure 1:**
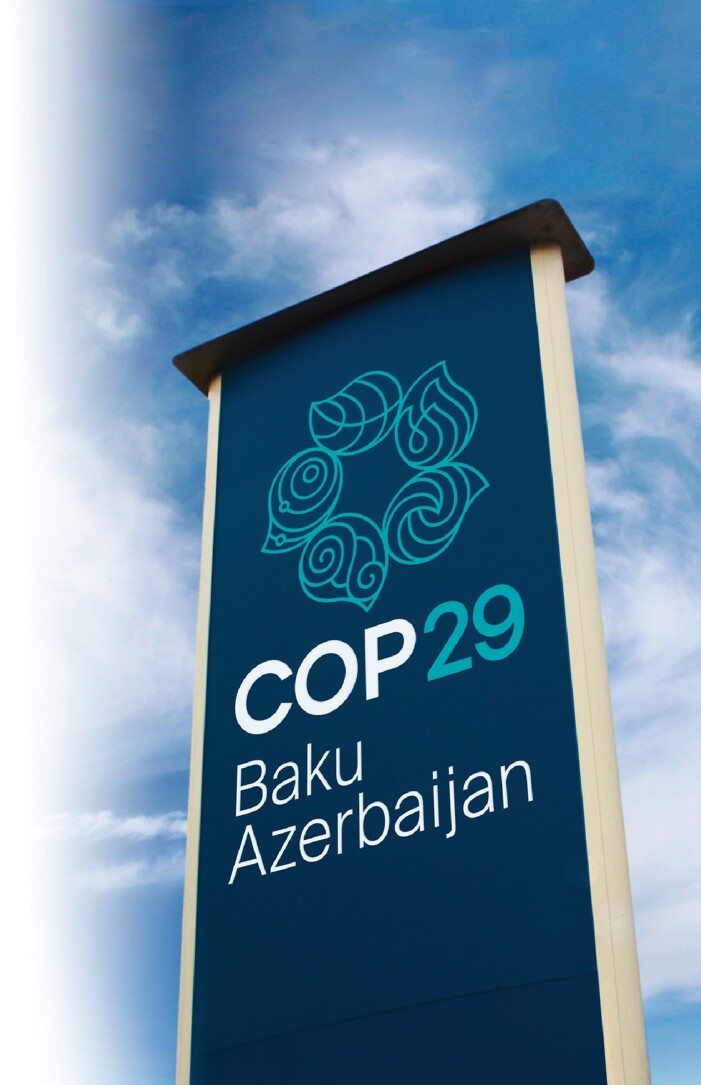
COP29 took place from 11 to 22 November 2024, in Baku, Azerbaijan. *(Photo: Adobe Stock)*

The year 2024 is another globally warmest year on record, continuing the uninterrupted sequence of 11 warmest years since 2014.

It is unequivocal that human influence has warmed the atmosphere, ocean and land. The 2024 Global Carbon Budget report [1] estimated that global CO_2_ emissions from fossil fuels reached a record high of 37.4 billion tons in 2024, 0.8% higher than that in 2023.

There is no sign that the world has reached a peak in fossil CO_2_ emissions, and the report estimates that at the current rate of emissions, there is a considerable (50%) chance that global warming will consistently exceed 1.5°C in ∼6 years. It means that the Paris Agreement goal, to keep a global temperature rise this century well below 2°C above the pre-industrial level and to pursue an even further goal of 1.5°C, is facing a great challenge, especially after President Trump pulled the USA out of this international climate change treaty on 20 January 2025, the first day of his second presidency term.

The Global Risks Report 2024 of the World Economic Forum (WEF, 2024) ranked the global risks by severity, with climate-related risks high on the list [2]. Extreme weather events and critical change to the Earth's systems were ranked as the first and second greatest risks for the long term (10 years), and the second and 11th greatest risks for the short term (2 years).

Facing all these challenges and risks, COP29 continued climate negotiations to construct a foundation for global climate actions. The conference reached a Baku Climate Unity Pact by adopting a series of important decisions (https://unfccc.int/cop29/auvs).

## NEW NATIONALLY DETERMINED CONTRIBUTIONS: ENHANCE AMBITION

The Nationally Determined Contributions (NDCs), being at the heart of the Paris Agreement [3] to be submitted every 5 years to the UNFCCC Secretariat, encapsulate the efforts of each country to reduce atmospheric greenhouse gas (GHG) emissions. Together, the total of national climate actions listed in the NDCs determines whether the world is likely to achieve the long-term goals of the Paris Agreement to curb GHG emissions.

‘It's time to deliver,’ said UN Secretary-General António Guterres to leaders and representatives gathered at COP29 in Baku. Indeed, representatives of Brazil and the United Kingdom delivered progressive and ambitious documents identifying the most recent NDCs, ahead of the deadline of 2025. By February 2025, all countries are required to submit new national climate plans or NDCs. This obligation is pushing nations to develop ambitious yet realistic NDCs that include private sector engagement.

Since the time to meet the Paris Agreement goals is rapidly running out, widespread and consistent climate action at the country level is needed. At the same time, developing country Parties that participated in the Climate Convention are allowed to continue increasing the emissions for some time for socio-economic grounds, with the understanding that emission reduction in these countries will start at a later time.

## A NEW CLIMATE FINANCE GOAL: ENABLE ACTION

The key outcome of COP29 is a new climate finance goal, which mainly aims to support the developing countries, including small island developing states, least developed countries, and African nations, to address the challenges posed by climate change.

During the COP15 held in Copenhagen in 2009, developed countries promised to provide $100 billion annually until 2020 in support of developing countries to cut emissions and better cope with the adverse effects of climate change. This commitment was later extended to 2025 under the Paris Agreement. However, it was not until 2022 that the developed countries first met that goal.

**Figure 2:**
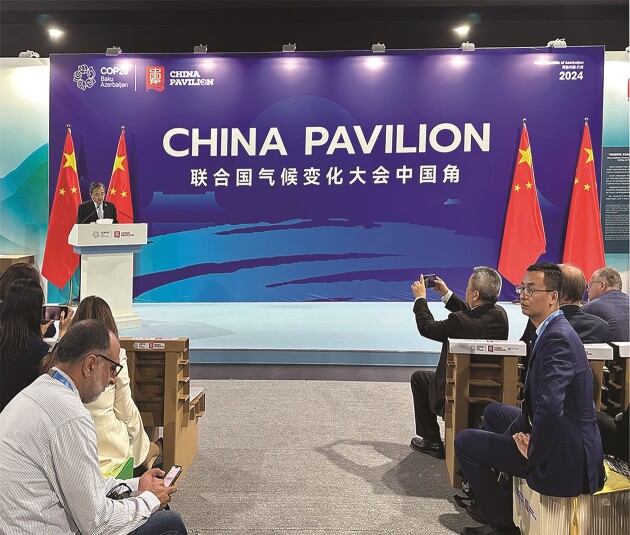
China Envoy for Climate Change Zhenmin Liu emphasized China's efforts and responsibilities at the COP29 China Pavilion. *(Photo: Tong Jiang)*

In accordance with the requirements of the Paris Agreement, a New Collective Quantitative Goal (NCQG) on climate finance for the period beyond 2025 had to be established, which is why COP29 is also referred to as the ‘Climate Finance COP’.

At COP29, the NCQG was agreed upon, obliging developed countries to provide at least $300 billion per year by 2035 to assist developing countries in adapting to climate change. The establishment of this goal marks a new phase in global climate finance and reflects the complexity and urgency of the financial issues in global climate governance.

Simon Stiell, the Executive Secretary of the United Nations Climate Change Secretariat, noted at the closing session that the new climate finance goal agreed at COP29 is ‘an insurance policy for humanity’, and ‘like any insurance policy—it only works—if the premiums are paid in full, and on time.’ The achievement of these goals requires the developed countries to take their responsibility and make more practical actions.

## THE WORLD'S EXPECTATIONS

The COP29 Conference set a spectacular 3-fold target increase from the climate finance goal set in 2009. However, a big gap still exists between the NCQG on Climate Finance and the amount actually needed by developing countries. Thus, further vigorous and persistent efforts are needed to meet the Sustainable Development Goal #13 [4], that is, global climate action.

A strategic plan, ‘Baku to Belém Roadmap to 1.3T’, was agreed upon during COP29, aiming at scaling up climate finance to developing countries to at least $1.3 trillion per year by 2035 from all public and private sources (https://unfccc.int/cop29/auvs). The Roadmap is designed to support low GHG emissions and climate-resilient development pathways, as well as the implementation of the NDCs and national adaptation plans (NAPs).

Parties are called upon to enhance their enabling environments, nationally, to increase the climate financing. The need for public and grant-based resources and highly concessional finance, particularly for adaptation and responding to climate loss and damage in vulnerable developing countries, is recognized. Likewise, the importance of continued efforts to support just transitions across all sectors and thematic areas is recognized.

Furthermore, a decision was made to ensure the operationalization of the Fund for Responding to Loss and Damage (FRLD, established at COP27), also to support the developing countries.

The next opportunity for further global negotiation on climate actions is COP30, planned to be held in Belém, Brazil in November 2025.

## Notes

None declared.
